# Comparison between patient-specific implants and hand-bent plates in mandibular reconstruction

**DOI:** 10.1007/s00784-026-06875-y

**Published:** 2026-04-24

**Authors:** Max Lukas Linderkamp, Efthymios Papazacharias, Thomas Lewandowski, Stephan Alexander Bettag, Jörg Mast, Fritjof Lentge, Philipp Jehn, Michael-Tobias Neuhaus, Nils-Claudius Gellrich, Philippe Korn

**Affiliations:** 1 Department of Oral and Maxillofacial Surgery, Hannover Medical School, Carl-Neuberg-Str. 1, 30625 Hannover, Germany; 2 Department of Otorhinolaryngology at München Klinik Schwabing, Kölner Platz 1, 80804 München, Germany

**Keywords:** Patient specific implants, Patient specific medicine, Mandibular reconstruction, Post-ablative reconstruction, Hardware-related complications

## Abstract

**Objectives:**

Alloplastic reconstruction is often required following segmental mandibular resection (SMR). Hardware-associated complications, especially hardware fractures, almost invariably necessitate explantation. Recent advances in manufacturing techniques promise potential advantages over traditional methods, including increased fracture resistance. However, to date, there is no scientific evidence confirming whether these benefits translate to real-life clinical conditions.

**Materials and methods:**

A total of 210 patients underwent SMR followed by alloplastic reconstruction between 2007 and 2019 at Hannover Medical School. The indications for SMR were tumor (*n* = 208; *n* = 196 squamous cell carcinoma) and osteoradionecrosis (*n* = 2). The mean patient age was 60.61 years, with a range of 14 to 89 years. The cohort comprised 146 male patients (69.52%) and 64 female patients (30.48%). Of these, 113 patients underwent reconstruction using hand-bent reconstruction plates (RP), while 97 received patient-specific implants (PSI). The incidence of hardware-related complications (HRC) was compared between the RP and PSI groups.

**Results:**

HRC occurred in 72 cases (34.29%). Exposure of the implant accounted for 47 cases (22.38%) while fractures occurred in 18 cases (8.57%), showing no significant difference between PSI and RP. Time from diagnosis until resection and reconstruction was not delayed due to the use of PSI.

**Conclusions:**

The choice of alloplastic manufacturing method does not affect HRC rates. PSI is offering benefits beyond fracture resistance and does not delay SMR.

**Clinical relevance:**

HRC remain challenging and may additionally impair patients’ quality of life. In particular, hardware fractures often necessitate additional surgical interventions, underlining the need to identify the factors that contribute to their occurrence. To the best of the author’s knowledge, this is the largest cohort study comparing RP and PSI with respect to HRC.

## Introduction

Segmental mandibular resection (SMR) may be required in the context of trauma, neoplasms such as cysts, semi-malignant tumors including ameloblastoma, or malignant neoplasms such as oral squamous cell carcinoma (OSCC). Medication- (especially antiresorptive agents) or radiotherapy-induced osteonecrosis can also necessitate SMR [1]. Alloplastic or autologous reconstruction is mandatory as SMR without reconstruction leads to heavy aesthetic and functional impairments, significantly affecting patients’ quality of life [2–4].

For alloplastic reconstruction of osseous defects resulting from SMR, surgeons either use traditional, hand-bent prefabricated reconstruction plates (RP) or modern patient-specific implants (PSI) designed during virtual surgical planning based on preoperative three-dimensional imaging [5].

While RP are cold-bent, PSI are either primarily sintered (additive manufacturing) or milled (subtractive manufacturing) into the correct anatomical shape without material tension, leading to higher stress resistance and plate strength. Kasper et al. demonstrated a significant advantage of PSI (additive and subtractive manufacturing) over RP in vitro. Whilst RP fractured at 150–210 N (N) and 40,000-643,000 cycles, PSI did not fracture despite a strain of 2,000,000 cycles and approximately double the load (330 N), assuming PSI to suffer significantly fewer hardware fractures [6]. Accordingly, Rendenbach et al. found PSI to be stiffer than Miniplates or RP [7]. While Kasper et al. showed PSI to be non-fracturable in vitro, Telschow et al. reported on two cases of material fractures in vivo in the enviably titled study “Unbreakable?” [8]. In accordance with the findings of Kasper et al. and Telschow et al., Zhong et al. published a study demonstrating that PSI outperformed RP in finite element analysis (FEA), showing one-third greater stiffness under static compression and more than a 90% increase in life cycles under cyclic loading [9].

Theoretically, the primary advantage of RP over PSI is the significantly shorter preoperative time required due to the elimination of planning and manufacturing processes. As “time is crucial” [10] in the treatment of progressive malignant diseases, this is particularly beneficial for patients under high pressure to act. Due to the lower planning requirements and lower demands for specialized manufacturing techniques, RP are significantly less expensive than PSI, by approximately a factor of seven in Germany.

PSI offer a more precise fit and shall reduce overall operating time, as they do require fewer intraoperative adjustments [11, 12]. Taking the costs of surgery time into account, this results in comparable overall costs [13]. Another advantage is that, especially in mandibular reconstruction, the anatomical dimensions [1] and, particularly, the centric condyle position can be obtained/restored more easily when planning PSI based on three-dimensional images taken with centric occlusion compared to RP [14]. Anatomical challenges, such as the interference of teeth roots or the inferior alveolar nerve, can be considered when planning angulated screw holes to preserve critical structures [1]. As microvascular reconstruction remains the gold standard for treating SMR defects, and osseous reconstruction is mandatory for conventional dental rehabilitation, PSI offer design elements that could support backward planning [15]. Newer studies suggest that PSI materials, engineered with triply periodic minimal surface structures, may enhance cell adhesion. When used in primary osseous reconstruction, this could enable accelerated osseointegration and, in turn, reduce the risk of plate loosening for patients who may need (adjuvant) radiation [16].

Despite advances in technology and the introduction of new, more biomechanically adapted design features in PSI [17], mandibular reconstruction remains challenging due to the complex anatomy of the mandible, including its temporomandibular joints, and the high biomechanical demands for reconstruction regarding aesthetics, speech, swallowing, masticatory, and breathing function [2, 17, 18].

In the past, many considerations and assumptions have been made regarding mandibular reconstruction after SMR using PSI, often without scientific evidence. Goodson et al. stated that “the overall scientific quality of the evidence available is average at best, with clinical evidence predominantly low-level and at moderate-to-high risk of bias” [19]. The present study aimed to assess whether some of the proclaimed benefits of PSI - such as increased fracture resistance and reduced surgical time - can be supported by scientific evidence. In clinical practice, only 18% of surgeons base their decision regarding the type of reconstruction plate used (rigid/flexible / locking/non-locking) after a fibula free flap on scientific evidence [20]. Although this scenario differs from that addressed in the present study, it clearly underscores the need for robust scientific data to guide hardware selection in mandibular reconstruction.

## Materials and methods

All patients who underwent SMR and alloplastic reconstruction at the Department of Oral and Maxillofacial Surgery at Hannover Medical School (MHH) between 2007 and 2019 were included in the analysis. Patient data was obtained from medical records using the clinic’s medical information system and was collected until 1st January 2025.

Minimum inclusion requirements included at least one follow-up (FU) visit after resection and alloplastic reconstruction, and the availability of both preoperative and postoperative radiologic images.

The study population was divided into two groups: one consisting of patients reconstructed with traditional, hand-bent RP and the other comprising patients reconstructed using PSI. The RP were fabricated from titanium with a minimum thickness of 2.3 mm; In contrast, the titanium PSI had a minimum thickness of 2.5 mm (RP and PSI manufactured by KLS Martin-Group, Tuttlingen, Germany, and DePuy Synthes^®^, Raynham, MA, USA).

The analysis contrasted the RP and PSI groups based on factors such as indication for SMR, sex, age, medical history (preexisting conditions that affect wound healing like diabetes or bisphosphonate intake, past oncological history and regional oncological history (cancer in the head and neck area) defect localization, and defect classification according to Brown’s classification for mandibular defects [21], defect length and volume, smoking history, and pre- and postoperative radiation therapy. Smoking history distinguishes between patients who have never smoked, quit smoking, and continued smoking after surgery.

The study assessed the potential advantages of PSI in reducing postoperative complications, including hardware exposure, fractures, infections, loosening of implanted hardware, and wound healing disorders. Other factors, such as the duration of surgery, length of hospitalization, and the time from diagnosis to resection and alloplastic reconstruction, were also evaluated. It was analyzed whether the type of soft-tissue flap affected hardware-related complications (HRC). Time from implantation until hardware fracture was documented. The Eichner classification was used to compare occlusal loading patterns in patients with hardware fractures.

The FU period was documented until 1st January 2025 and was defined as the time interval between surgery and the last available clinical or radiologic assessment. Postoperative FU included both inpatient assessment and outpatient clinical examinations performed after hospital discharge. Upon the occurrence of a major event, such as explantation of the PSI/RP, secondary osseous reconstruction, dental implantation, recurrence, or the patient’s death, FU was discontinued, and the patient was no longer included in subsequent FU assessments. FU was also discontinued due to irregular attendance at scheduled appointments or patients’ preference for local FU care.

In case of partially missing data, missing values were omitted from the corresponding statistical analysis, while the remaining data from the cases were included.

Statistical significance was determined using the chi-square (χ²), Fisher’s exact and t-test. The Bonferroni correction was applied to exclude falsely significant results arising from multiple simultaneous hypothesis testing. A binary logistic regression analysis was performed to minimize confounding, particularly with respect to smoking status and HRC amongst the PSI and RP groups. Model fit was assessed using the Hosmer-Lemeshow (HL) test. A result > 0.05 indicated a good fit of the model to the dataset. Kaplan-Meier survival analysis and a log-rank test were performed to evaluate the association between hardware type and the occurrence of hardware fractures. Statistical analyses were conducted using Microsoft Excel 2016 (Microsoft, Redmond, WA, USA) and IBM SPSS Statistics^®^ Version 30.0.0.0 (IBM, Armonk, NY, USA). A *p*-value of ≤ 0.05 was considered statistically significant.

## Results

### Research collective

Between 2007 and 2019, 210 patients underwent SMR followed by alloplastic reconstruction at the Department of Oral and Maxillofacial Surgery at MHH. Of these, 113 patients (53.81%) received RP, while 97 (46.19%) received PSI.

The primary indications for SMR were tumors (*n* = 208, 99.05%; SCC accounting for 196 (93.33%), osteosarcoma for four (1.90%), and other entities for eight cases (3.81%)) and osteonecrosis (*n* = 2, 0.95%). The mean patient age was 60.61 years (SD = 12.15, range: 14–89 years). The cohort included 146 male patients (69.52%) and 64 female patients (30.48%).

No significant differences were observed between the analyzed cohorts, except for smoking status. A significantly higher proportion of patients reconstructed with PSI had quit smoking, while fewer had never smoked (*p* [χ²] = 0.015) (Table 1). Post-hoc analysis showed no significant difference in the distribution of never smokers and persons who continued smoking between the two cohorts (Bonferroni post-hoc *p* = 1.000, 95% CI [-0.46, 0.25]).

The type of reconstruction hardware used was not significantly associated with SMR defect localization according to Brown’s-classification (*p* = 0.702), length or volume (Length: *p* [t-Test] = 0.247; RP: mean = 62.67 mm, SD = 19.63, range: 29–123 mm; PSI: mean = 66.60 mm, SD = 23.92, range: 33–144 mm. Volume: *p* [t-Test] = 0.802; RP: mean = 15.79 cm³, SD = 11.69, range: 4.15–103.32 cm³; PSI: mean = 15.43 cm³, SD = 7.10, range: 5.38–37.49 cm³).

**Table 1:** Demographic and clinical features of the patients analyzed, *p* [χ²] showing statistically significant differences between the cohorts reconstructed using RP and PSI

Feature	RP (*n*)	%	PSI (*n*)	%	*p* [χ²]
Diagnosis					0.914
Tumor	112	99.12	96	98.97	
Osteoradionecrosis	1	0.88	1	1.03	
Sex					**0.639**
Male	77	68.14	69	71.13	
Female	36	31.86	28	28.87	
Age					**0.669**
< 30 years	3	2.65	2	2.06	
30 - < 50 years	17	15.04	10	10.31	
50 - < 70 years	65	57.52	63	64.95	
70 - < 90 years	28	24.77	22	22.68	
Pre-existing conditions					
Diabetes	14	12.39	10	10.31	**0.798**
Bisphosphonate intake	1	0.88	3	3.03	**0.508**
Past-oncological history (POH)					**0.425**
Breast cancer	4	3.54	4	4.04	
Prostate cancer	3	2.65	0	0.00	
Colorectal cancer	2	1.77	1	1.01	
Other	3	2.65	5	5.05	
Regional POH					**0.663**
Oral cavity and oropharynx	20	17.70	19	19.59	
Other	3	2.65	1	1.01	
Localization of the defect					**0.476**
Lateral	74	65.49	68	70.10	
Anterior	39	34.51	29	29.90	
Brown-Classification					0.710*
I	39	34.51	31	31.96	
Ic	4	3.54	8	8.25	
II	26	23.01	25	25.77	
IIc	3	2.65	3	3.09	
III	30	26.55	21	21.65	
IV	6	5.31	5	5.15	
IVc	2	1.77	4	4.12	
Missing	3	2.65	0	0.00	
Smoking					**0.015**
Never	42	37.17	28	28.87	
Former	4	3.54	14	14.43	
Continued	67	59.29	55	56.70	
Radiatio (pre- and postoperative)					**0.976**
Yes	72	63.72	62	63.92	
No	41	36.28	35	36.08	
Radiatio (preoperative)					**0.064**
Yes	25	22.12	12	12.37	
No	88	77.88	85	87.63	
Total	**113**	**53.30**	**97**	**46.70**	

* *p* was calculated excluding the missing data and using Fisher’s exact test

### Hardware-related complications

A total of 72 HRC occurred, representing 34.29% of cases. The most common complication (65.28% of HRCs) was implant exposure, accounting for 47 cases. Hardware fractures were observed in 18 cases (8.57% overall, 25.00% of HRC) and occurred independently of the used alloplastic approach (*p* [χ²] = 0.735). In case of hardware fractures the Eichner classification did not differ significantly between the two cohorts (*p* [Fisher] = 0.720). Overall, no significant difference was observed between the two cohorts (Table 2). However, a slight, non-significant difference was noted in implant infections (II), with patient-specific implants (PSI) marginally less affected (*p* [Fisher] = 0.182). II was observed mainly in smokers receiving RP (6 of 9 II, *p* [χ²] = 0.092). Overall, no difference was observed between RP and PSI for HRC (p [χ²] = 0.940) or the need for hardware removal (*p* [χ²] = 0.248).

**Table 2:** HRC using RP and PSI

Feature	RP (*n*)	%	PSI (*n*)	%	*p* [χ²]
Exposure					0.668
Yes	24	21.24	23	23.71	
No	89	78.76	74	76.29	
Fracture					**0.735**
Yes	9	7.96	9	9.28	
No	104	92.04	88	90.72	
Eichner classification in case of hardware fracture (*n* = 18)					0.720*
B1	2	22.22	1	11.11	
B2	3	33.33	6	66.67	
B3	1	11.11	1	11.11	
B4	1	11.11	0	0.00	
C3	2	22.22	1	11.11	
Infection					0.182*
Yes	7	6.19	2	2.06	
No	106	93.81	95	97.94	
Loosening					0.538*
Yes	1	0.88	0	0.00	
No	112	99.12	97	100.00	
Wound infection					0.229*
Yes	8	7.08	3	3.09	
No	105	92.92	94	96.91	
Total	**113**		**97**		

*p was calculated using Fisher’s exact test

Primary soft-tissue reconstruction was performed using a latissimus dorsi flap (*n* = 91, 43.33%), a radial forearm flap (*n* = 60, 28.57%), or a lateral upper arm flap (*n* = 25, 11.90%) in the majority of cases, whereas other regional flaps (*n* = 12, 5.71%) and local flaps (*n* = 9, 4.29%) were used rarely. Local reconstruction was performed in the minority of cases (*n* = 13, 6.19%). There was no statistically significant association between the type of soft-tissue reconstruction and the occurrence of HRC (p [χ²] = 0.588). In particular, no significant correlation was found between hardware exposure or II and the type of primary soft-tissue reconstruction (p [χ²] = 0.294; p [χ²] = 0.101, respectively). There was no significant correlation between preoperative or pre- and postoperative radiation and hardware exposure (*p* [χ²] = 0.755; *p* [χ²] = 0.300, respectively).

A binomial logistic regression was performed to determine whether the smoking status and the type of reconstruction plate used predict the likelihood of hardware fractures and hardware exposure. Regarding hardware fractures Hosmer-Lemeshow-Test indicating a good model fit, χ²(3) = 3.236, *p* = 0.357. None of the variables entered into the regression model contributed significantly to predicting hardware fractures, Exp(B_former smokers_) = 1.754, 95% CI [0.39, 7.96], *p* = 0.466; Exp(B_smokers_) = 0.629, 95% CI [0.22, 1.82], *p* = 0.392; Exp(B_PSI_) = 1.071, 95% CI [0.39, 2.92], *p* = 0.894.

Concerning hardware exposure, former smokers are affected significantly more often (*p* = 0.001), regardless of the hardware used (see Table 3).

**Table 3:** Binomial logistic regression model determining whether the smoking status and the used hardware for alloplastic reconstruction predict the likelihood of hardware exposure. Reference categories are *never smokers* and *RP*

Feature	χ²(df)	Exp (B)	95% CI	*p*
Plate exposure				
HL	0.648 (3)			0.885
Former smoker		6.998	2.128, 23.014	0.001
Smoker		2.123	0.939, 4.798	0.070
PSI		0.920	0.462, 1.834	0.813

### Time aspects

The average duration of surgical procedures between the RP and PSI groups did not differ significantly (*p* [t-Test] = 0.627). On average, patients were hospitalized for 26.60 days (*n* = 209, SD = 12.38, range: 10–95 days), regardless of the reconstruction method used (*p* [t-Test] = 0.818).

The mean time from diagnosis to resection and implantation of RP/PSI was 36.08 days (*n* = 210, SD = 29.10, range: 5-342 days), with no significant difference between the RP and PSI groups (*p* [t-Test] = 0.686). Reconstruction could be performed as early as five days following diagnosis with both RP and PSI.

### Follow-up

Mean FU was 544.59 days (*n* = 210, SD = 588.30, range: 15-3387 days). FU periods for the RP group (462.59 days, SD = 553.46, range: 22-3014 days) are comparable to the PSI group (447.42 days, SD = 629.27, range: 15-3387 days; *p* [t-Test] = 0.854).

**Fig. 1:**
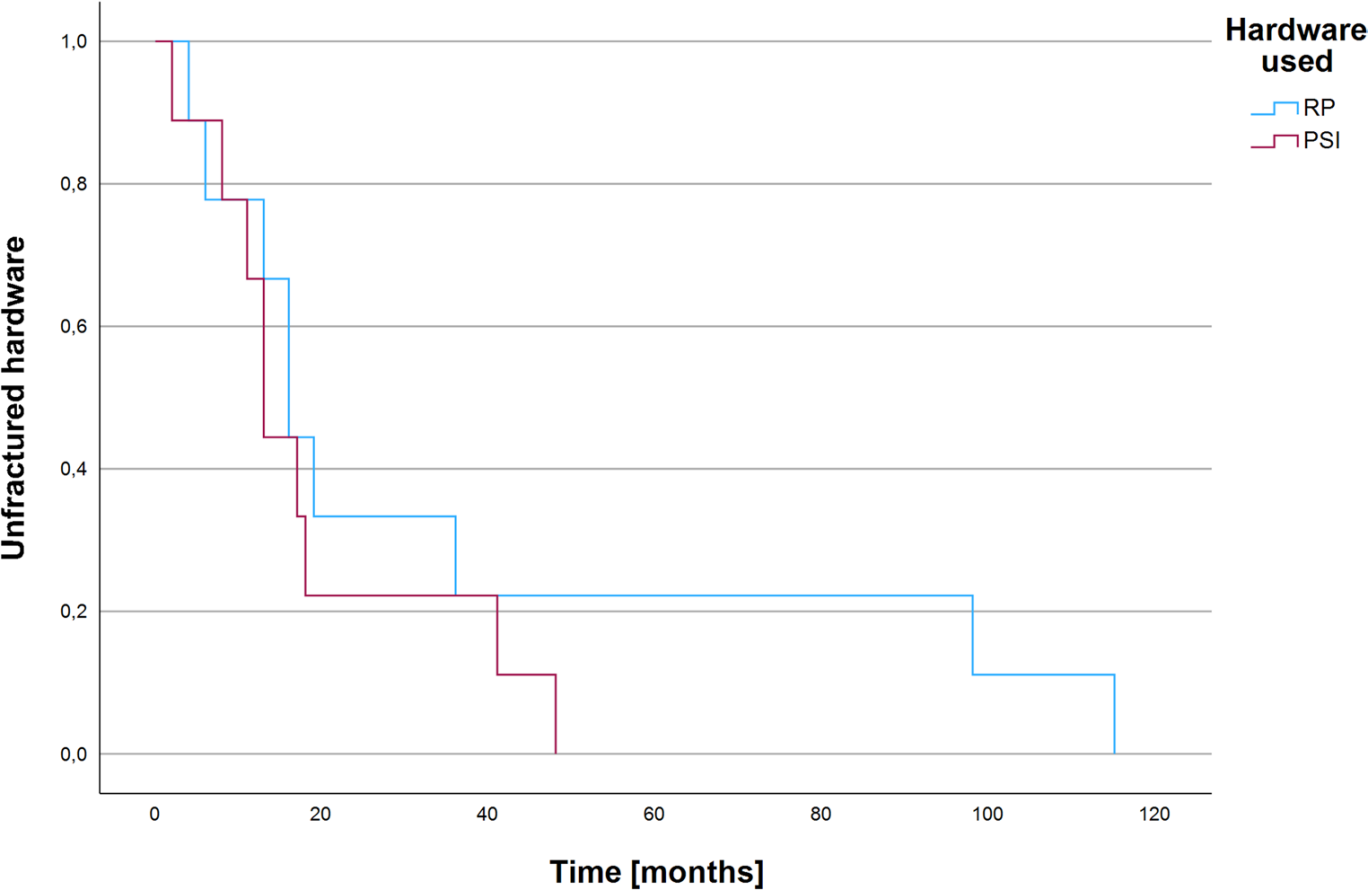
Kaplan-Meier analysis showing no significant difference regarding time from implantation until hardware fracture between the PSI and the RP group (each *n* = 9)

Mean time from implantation until hardware fracture was 27.44 months (*n* = 18, SD = 31.42, range: 2-115 months). Hardware fracture occurred faster in PSI (*n* = 9, mean = 19.00 months, SD = 15.31, range: 2–48 months) compared to RP group (*n* = 9, mean = 35.89 months, SD = 41.27, range: 4-115 months), without significance (*p* [t-Test] = 0.276 and log-rank-test: χ²(1) = 0.885, *p* = 0.347, see Fig. 1).

## Discussion

**Fig. 2:**
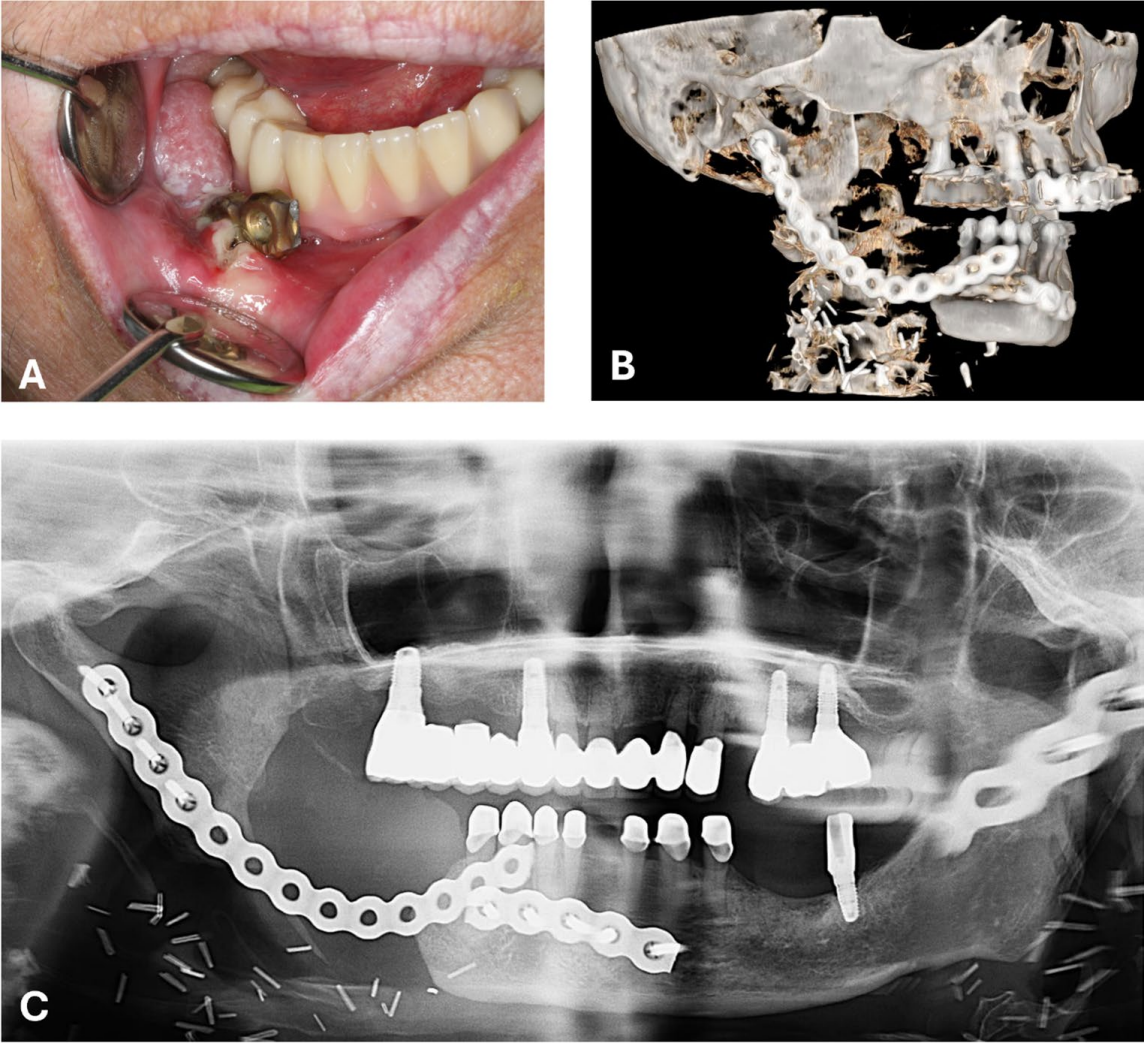
Fractured RP, penetrating the oral cavity, underlining the necessity for removal (**A**), radiographic images (**B**: virtual reconstruction of a Cone Beam Computed Tomography (CBCT), **C**: panoramic radiograph) of the same patient

Hardware fractures occurred in about 8.6% of cases, which is comparable to complication rates reported within the current literature [22] and remain one of the most challenging problems in alloplastic mandibular reconstruction, often necessitating implant explantation (Fig. 2). Whilst Zeller et al. have shown that RP have presented with significantly more hardware fractures (RP = 17%; PSI = 0%, *n* = 42, of which 30 PSI, *p* = 0.022) [14], the present study, including significantly more patients, could not replicate their findings.

**Fig. 3:**
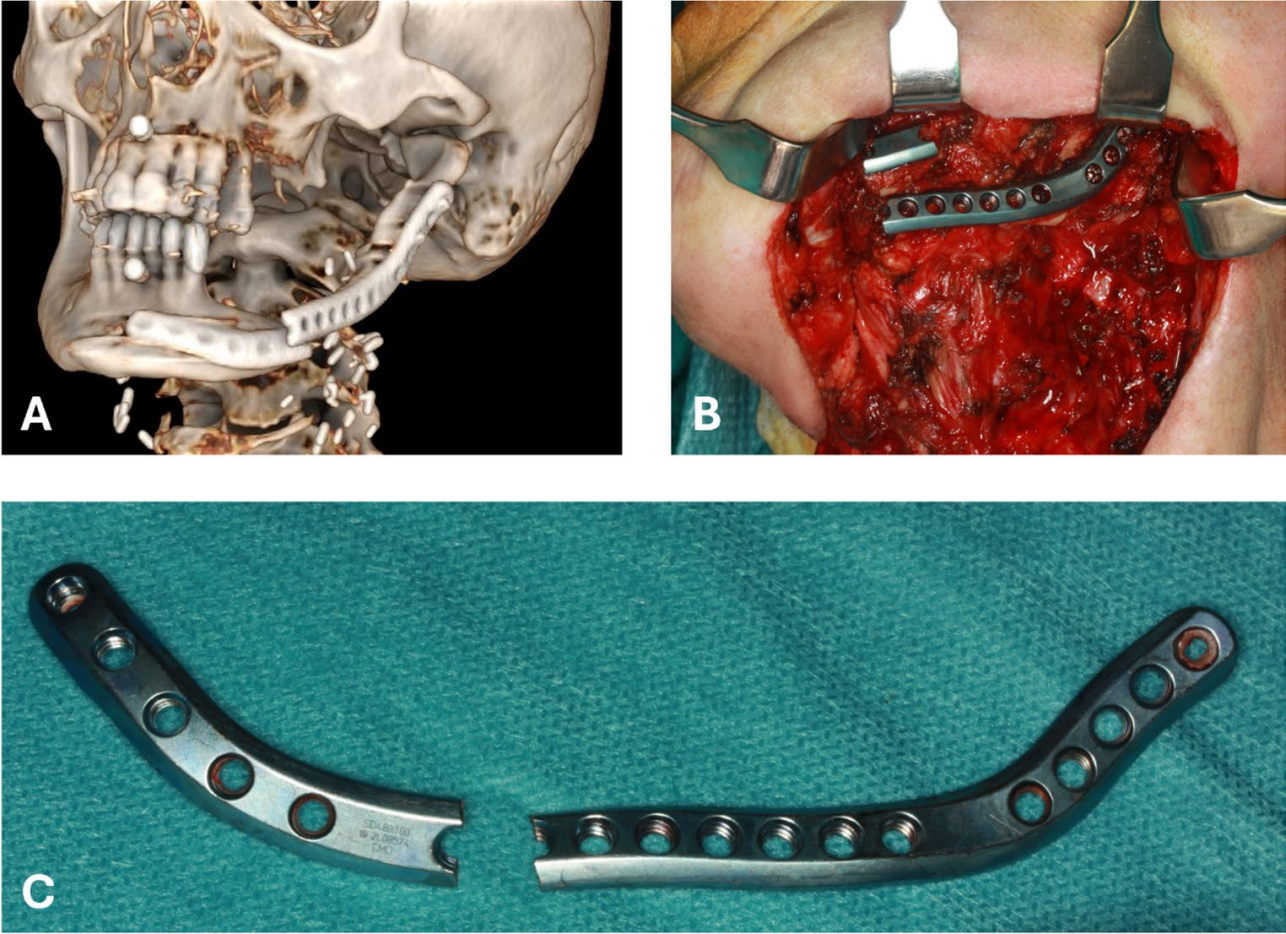
Fractured PSI in virtual reconstruction of a CBCT (**A**), intraoperative pictures in situ (**B**), and after explantation (**C**)

Whereas in vitro experiments showed PSI to be superior to RP in terms of fracture resistance [6, 7, 9] this could not be proven in vivo (Fig. 3). This demonstrates the complex biomechanical function of the mandible and the challenges of translating in vitro findings into real-world applications. Goodson et al. stated that “complication rates with this novel approach [PSI] appear to be in keeping with existing / traditional techniques” whilst also having an “increasingly valuable role in mandibular reconstruction” [19]. This underlines that, to date, the benefits of PSI appear to lie more in functional outcomes than in fracture resistance.

As a retrospective study this study has several limitations that are characteristic of retrospective surgical-clinical research. First, although board-certified tumor surgeons performed all procedures at an accredited German tumor center, the use of RP or PSI was not randomized.

Second, the extended study period introduces the potential for period bias. Over time, both the operating surgeons and the treatment algorithms changed. The prolonged observation period particularly affected the devices’ technical characteristics, as PSI are subject to continuous modification. Consequently, PSI design was not uniform across the study period. In addition, non-surgical factors - such as temporal changes in evidence-based antibiotic protocols adopted at different stages of the study - may also have influenced the observed outcomes.

Despite these limitations, the study benefits from a large cohort, which, to the best of the authors’ knowledge, is the largest reported to date to investigate HRC of PSI and RP in mandibular reconstruction. Furthermore, the data were generated at a high-volume, experienced, and certified tumor center.

In the present study, FU was terminated upon the occurrence of a major event. In most cases, RP/PSI were removed during secondary osseous reconstruction. Even if RP/PSI remained in situ, FU was discontinued at that time, as the implanted bone plays a critical role in mandibular stability and would therefore confound the results, particularly with respect to hardware fractures.

Although the total operative time did not differ significantly between the two groups (RP vs. PSI), the effect of the reconstructive hardware used on operative duration is difficult to quantify. It is likely masked by the overall length of the surgical procedure. The interval between completion of the resection and final implantation of RP or PSI would represent a more appropriate measure; however, this variable is not available due to the study’s retrospective design.

In the present study, most patients received distant flap reconstruction for primary soft-tissue reconstruction. The choice of flap did not significantly affect HRC. Primary soft-tissue coverage plays a vital role in mandibular reconstruction, not only for HRC, but also for functional outcomes [23].

Regarding the background of higher costs and the need for preoperative determination of resection margins when using PSI, RP should nevertheless be considered in daily clinical practice, particularly when fast and durable alloplastic reconstruction strategies are required.

Contrary to previous assumptions, the present study does not demonstrate any difference in the time from diagnosis to hardware implantation or in the duration of surgery between PSI and RP. The use of PSI does not delay resection and reconstruction in the investigated cohort. Despite the increased production time, other organizational factors, such as operating theatre capacity, appear to play a more significant role, suggesting no noticeable loss of time for the patient.

It should be noted that PSI, unlike RP, may be better able to restore the centric condyle position through preoperative planning and design, thereby preventing postoperative complications such as craniomandibular dysfunction or temporomandibular joint arthrosis. This idea is derived from orthognathic surgery, where Bill et al. demonstrated that continuous centric condyle positioning leads to a significantly better functional outcome (*p* < 0.05) [24].

Whilst no advantage in fracture probability could be shown, the use of PSI could offer further benefits. Design features in PSI could be helpful in cases with two-stage reconstruction and potential osseous free flap reconstruction [25]. More complex designs, not suitable for RP, could help with easier protection of the inferior alveolar nerve [1]. A major advantage has been demonstrated in three-dimensional reconstruction of the mandible with implant-predetermined segment positioning, leading to lower mandibular segment dislocations (*p* < 0.05) [17] and higher mandibular reconstruction accuracy [14].

**Fig. 4:**
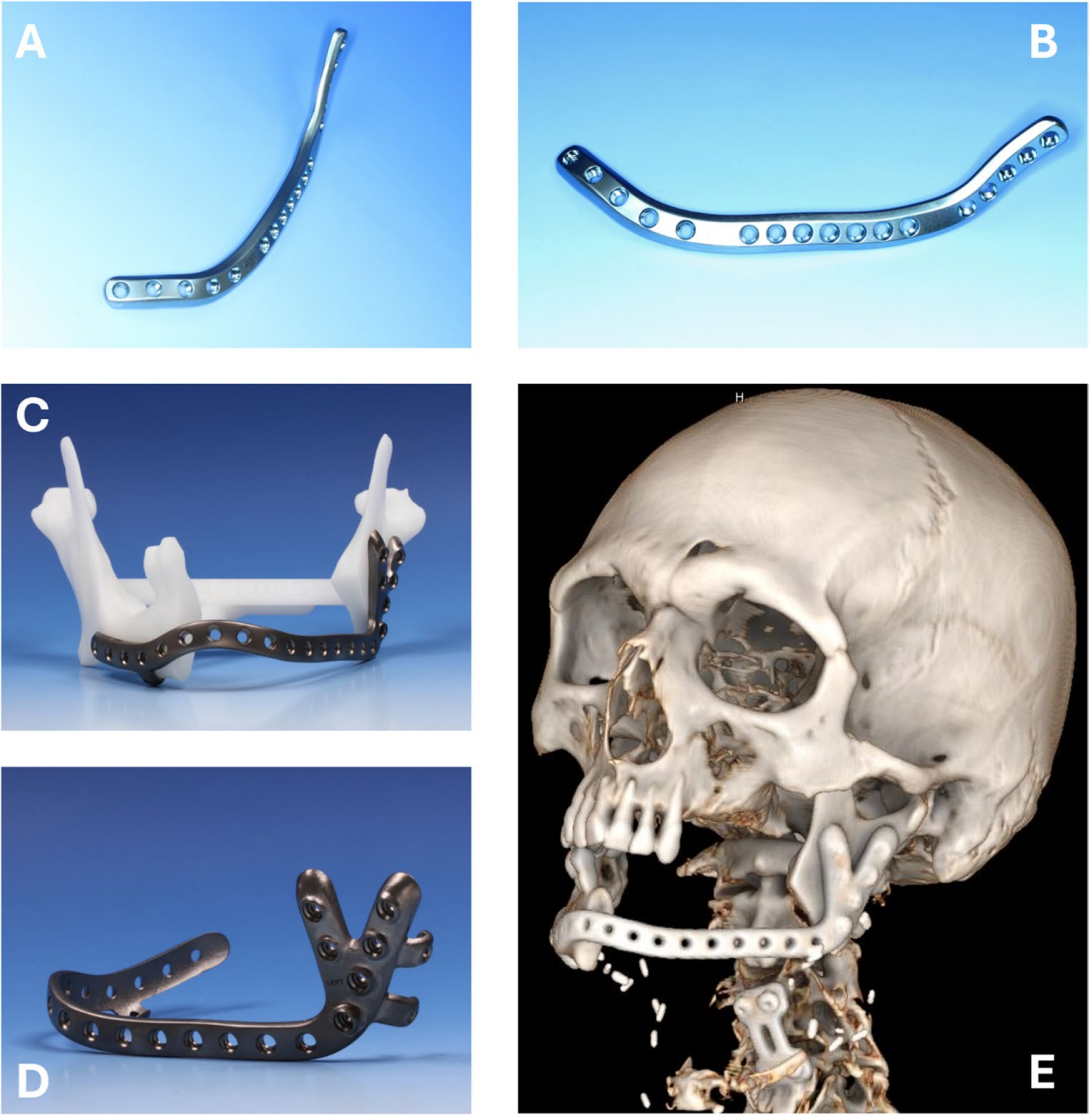
Comparison between first (**A** and **B**) and second-generation PSI (**C**, **D**, and **E**). Second-generation PSI offering design benefits for one-fit-only application; frontal (**A**, **C**) and lateral view (**B**, **D**), and after implantation in virtual reconstruction of a computed tomography (**E**)

Since most of the examined PSI were implanted before 2017, it can be assumed that the majority belongs to the first-generation PSI manufactured in a regular plate design (e.g., lacking Y-shape design and three-dimensional, multivectorial one-fit anchorage, Fig. 4). In contrast, PSI of the second generation (introduced in 2017) allows for a more precise reconstruction of anatomical dimensions and better prevention of centric condyle position [26]. Due to the more complex design of second-generation PSI, a one-way fit may reduce biomechanical stress on PSI and, consequently, lead to fewer fractures compared to first-generation PSI. However, this remains an assumption that requires further clinical research for validation. FEA, as is usually used for other implant types such as subperiosteal implants during production, may also help simulate material hardware strain for PSI, thereby preventing fractures [27, 28].

To the best of the author’s knowledge, this is the largest cohort study comparing RP and PSI, particularly regarding fracture resistance and HRC, and shows no difference in the likelihood of hardware fractures. But: The current literature states that PSI offers benefits beyond fracture resistance.

## Data Availability

The data that support the findings of this study are not openly available due to reasons of sensitivity and are available from the corresponding author upon reasonable request. Data are located in controlled access data storage at MHH.
